# Inhibitory action of phenothiazinium dyes against *Neospora caninum*

**DOI:** 10.1038/s41598-020-64454-x

**Published:** 2020-05-04

**Authors:** Luiz Miguel Pereira, Caroline Martins Mota, Luciana Baroni, Cássia Mariana Bronzon da Costa, Jade Cabestre Venancio Brochi, Mark Wainwright, Tiago Wilson Patriarca Mineo, Gilberto Úbida Leite Braga, Ana Patrícia Yatsuda

**Affiliations:** 1 Faculdade de Ciências Farmacêuticas de Ribeirão Preto, Universidade de São Paulo, Av do Café, sn/n, 14040-903 Ribeirão Preto, SP Brazil; 2 Núcleo de Apoio à Pesquisa em Produtos Naturais e Sintéticos, Universidade de São Paulo, Ribeirão Preto, SP Brazil; 3 Department of Immunology, Institute of Biomedical Sciences, Federal University of Uberlândia, Uberlândia, Brazil; 4 School of Pharmacy and Biomolecular Sciences, Liverpool John Moores University, Liverpool, L3 3AF United Kingdom

**Keywords:** Parasite development, Parasitic infection

## Abstract

*Neospora caninum* is an Apicomplexan parasite related to important losses in livestock, causing abortions and decreased fertility in affected cows. Several chemotherapeutic strategies have been developed for disease control; however, no commercial treatment is available. Among the candidate drugs against neosporosis, phenothiazinium dyes, offer a low cost-efficient approach to parasite control. We report the anti-parasitic effects of the phenothiaziums Methylene Blue (MB), New Methylene Blue (NMB), 1,9–Dimethyl Methylene Blue (DMMB) and Toluidine Blue O (TBO) on *N. caninum*, using *in vitro* and *in vivo* models. The dyes inhibited parasite proliferation at nanomolar concentrations (0.019–1.83 μM) and a synergistic effect was achieved when Methylene Blue was combined with New Methylene Blue (Combination Index = 0.84). Moreover, the phenothiazinium dyes improved parasite clearance when combined with Pyrimethamine (Pyr). Combination of Methylene Blue + 1,9–Dimethyl Methylene Blue demonstrated superior efficacy compared to Pyrimethamine based counterparts in an *in vivo* model of infection. We also observed that Methylene Blue, New Methylene Blue and 1,9–Dimethyl Methylene Blue increased by 5000% the reactive oxygen species (ROS) levels in *N. caninum* tachyzoites. Phenothiazinium dyes represent an accessible group of candidates with the potential to compound future formulations for neosporosis control.

## Introduction

*Neospora caninum*, an obligate intracellular protozoan, belongs to Apicomplexa phylum, such as *Plasmodium* spp and *Toxoplasma gondii*. It causes neosporosis, a disease strongly correlated to abortion and decreased fertility in cattle, costing billions of dollars worldwide^1–3^. There is no effective commercial treatment for neosporosis, despite the efforts of several chemotherapy-focused groups^4–6^. For example, Artemisinins^7–10^, Miltefosine^11^, plant extracts^12–14^, Diamidines^15,16^, Buparvaquone^4,17^, organometallic ruthenium complexes^18^, Thiazolides^19,20^ were evaluated in *in vitro* and *in vivo* models with encouraging results. Moreover, polyether ionophore antibiotics^21^, Triazinones^22–24^, bumped kinase inhibitors^25^ have also been tested in farm ruminants^26^.

Based on the promising effects of Methylene Blue (MB) against *Plasmodium* spp, the etiologic agent of malaria, our group determined the efficacy of this molecule on *N. caninum*, either alone or combined with Pyrimethamine (Pyr)^27^. MB, a phenothiazinium dye, was the first synthetic drug described to cure a patient affected by malaria in the XIX century, pioneering work performed by Paul Ehrlich^28,29^. Indeed, MB was used against *Plasmodium* until the use of Chloroquine and other drugs (massively utilized after the Second World War), which lack some of the reversible MB side effects (blue urine and sclera)^30,31^. During the later 20^th^/early 21^st^ Centuries, the intense use of Chloroquine, Artemisinin, and Pyrimethamine has led to proven cases of *Plasmodium* resistance, demanding novel therapeutic candidates^32^. Interestingly, no reports of *Plasmodium* resistance have been reported in experimental MB-based therapies, reviving the dye as an antimalarial drug^33^. Moreover, phenothiazinium dyes such as New Methylene blue and Toluidine Blue O also demonstrated antimicrobial properties^34,35^, inspiring us to test these compounds against *N. caninum*.

Ehrlich’s brilliant observations, concerning Methylene Blue suggest that other phenothiazinium derivatives (New Methylene Blue, Toluidine Blue O, and 1,9-Dimethyl Methylene Blue) may also provide safe, low-cost candidates to treat parasitic diseases. Since the livestock industry is particularly sensitive to costs, inexpensive formulations based on phenothiazinium dyes have the potential to improve the outcomes in cattle affected by neosporosis. To this end, we have investigated the anti-parasitic effects of the phenothiaziums Methylene Blue, New Methylene Blue, 1,9-Dimethyl Methylene Blue and Toluidine Blue O on *N. caninum*, using *in vitro* and *in vivo* models.

## Results and discussion

### Proliferation and combination assays

MB is the most commonly employed phenothiazinium dye, with therapeutic potential against malaria both alone and in associations with Artemisinin, Quinine, Chloroquine and Pyrimethamine^33,36^. MB inhibits *N. caninum* proliferation, with an IC_50_ of 0.349 μM (^27^, Table 1). NMB, TBO, and DMMB are Methylene Blue derivatives with diverse modifications (Table 1). For example, in NMB, the dimethylamino- groups of MB are substituted by ethylamino- moieties and two additional methyl groups are inserted in the phenothiazinium core next to the ethylamino groups. TBO has one dimethylamino- and one amino group, with a methyl group next to the latter. DMMB has the same structure as MB, but with two methyl groups in the positions on either side of the ring nitrogen. NMB, DMMB, and TBO inhibited *N. caninum* proliferation at IC_50_ values of 0.058 μM, 0.019 μM and 1.83 μM respectively (Table 1).

**Table 1:** *In vitro* IC_50_ and toxicity of MB, NMB, DMMB, and TBO to *N. caninum* and Vero cells.

Name	Structure	MW	IC_50_ μM	CC_50_ μM	SI
MB		319.85	0.349 *	>62.5 *	>179.1*
NMB		347.91	0.048 ± 0.012	21.893 ± 7.851	456.1
DMMB		416.05	0.019 ± 0.004	8.524 ± 3.317	448.6
TBO		305.83	1.83 ± 0.124	>25	>13.6

The IC_50_, CC_50,_ and selectivity index were calculated for NMB, DMMB, and TBO on *N. caninum* and Vero cells. Tachyzoites or Vero cells were incubated for 72 h, at 37 °C, with 5% CO_2_, and the proliferation (tachyzoites) or toxicity (Vero cells) was measured after CRPG or MTT assays respectively. The percentage of inhibition was calculated in comparison to the non-treated controls in three independent assays. *^27^.

**Figure 1:**
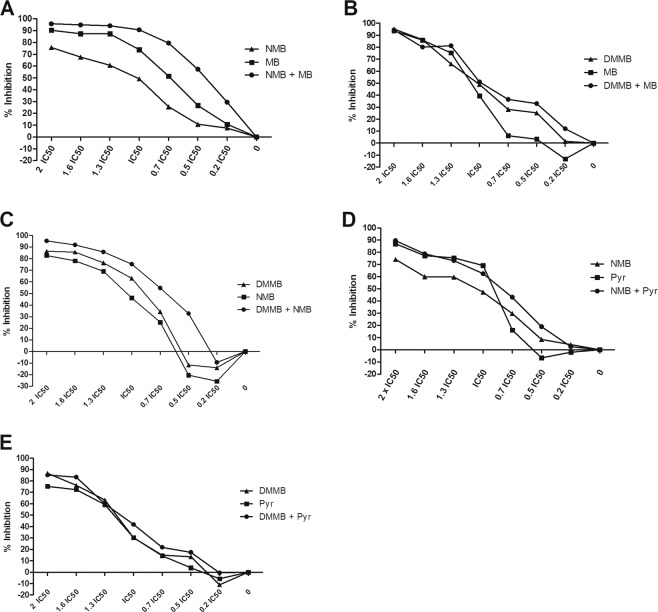
Inhibitory activity of phenothiazinium dye combinations against *N. caninum*. Tachyzoites were incubated with seven dilutions (2 × IC_50_, 1.6 × IC_50_, 1.3 × IC_50_, IC_50_, 0.7 × IC_50_, 0.5 × IC_50_, 0.2 × IC_50_) of NMB + MB (**A**), DMMB + MB (**B**), DMMB + NMB (**C**), NMB + Pyr (**D**) and DMMB + Pyr (**E**) and the proliferation measured after CPRG assay. As references, isolated compounds of each combination were evaluated concomitantly in the same plate. The inhibition percentage was calculated in comparison to the non-treated group and the IC_50_ and CI values were achieved using Compusyn software.

The combination of MB with NMB demonstrated a synergistic effect. The IC_50_ of MB and NMB decreased from 0.221 ± 0.048 μM to 0.119 ± 0.05 μM and from 0.069 ± 0.006 μM to 0.021 ± 0.008 μM, respectively, with a CI of 0.84 (Fig. 1A; Table 2). However, the combination of MB and DMMB demonstrated an antagonistic effect (CI = 1.36), despite the improvement of the inhibitory pattern. The IC_50_ of MB diminished from 0.394 ± 0.109 μM to 0.249 ± 0.146 μM and of DMMB from 0.024 ± 0.013 μM to 0.017 ± 0.012 μM (Fig. 1B; Table 2). Although a low IC_50_ was observed when NMB or DMMB was applied alone (Table 1), the combination between these compounds was also antagonistic (CI = 1.40). The doses of NMB and DMMB were altered from 0.064 ± 0.018 μM and 0.021 ± 0.002 μM to 0.040 ± 0.004 μM and 0.016 ± 0.002 μM respectively (Fig. 1C; Table 2). All Pyr combinations were antagonistic (Fig. 1D,E). The Pyr IC_50_ combined with NMB decreased from 0.347 ± 0.044 μM to 0.301 ± 0.077 μM whereas NMB decreased 0.072 ± 0.012 μM to 0.053 ± 0.018 μM, with a CI of 1.60 (Fig. 1D; Table 2). When Pyr was combined with DMMB (CI = 1.78) the IC_50_ doses decreased from 0.436 ± 0.184 μM to 0.377 ± 0.159 μM and from 0.029 ± 0.012 μM to 0.026 ± 0.016 μM, respectively (Fig. 1E; Table 2). Due to its higher inhibitory concentration (1.83 μM), TBO was not tested in combination assays. The combinations based on phenothiazinium dyes demonstrated different patterns of efficiency, which indicates that their mechanisms of action might be different and the molecules might interact or accumulate differently when combined. Therefore, novel assays concerning the mechanisms of action of phenothiazinium dyes in *N. caninum* will be required in future approaches. Although Pyr is a promising candidate against *N. caninum*^6,27^, the NMB, MB and DMMB combinations were superior when compared to Pyr counterparts in proliferation assays.

**Table 2:** Values of inhibitory concentrations (IC_50_) and Combinatory Index of the phenothiazinium combinations.

Combination (compound 1 + 2)	IC_50_ (μM) (compound 1)	IC_50_ (μM) (compound 2)	IC_50_ (μM) (combination in relation to compound 1)	IC_50_ (μM) (combination in relation to compound 2)	CI
NMB + MB	0.069 ± 0.006	0.221 ± 0.048	0.021 ± 0.008	0.119 ± 0.05	0.84
DMMB + MB	0.024 ± 0.013	0.394 ± 0.109	0.017 ± 0.012	0.249 ± 0.146	1.36
NMB + DMMB	0.021 ± 0.002	0.064 ± 0.018	0.016 ± 0.002	0.040 ± 0.004	1.40
NMB + Pyr	0.072 ± 0.012	0.347 ± 0.044	0.053 ± 0.018	0.301 ± 0.077	1.60
DMMB + Pyr	0.029 ± 0.012	0.436 ± 0.184	0.026 ± 0.016	0.377 ± 0.159	1.78

The IC_50_ concentrations of the dyes alone and in combinations were calculated using the Compusyn software^66^. The software was also used for the determination of the CI between the combined compounds.

### Dyes and Pyrimethamine induces resistance in parasites under selection

**Figure 2:**
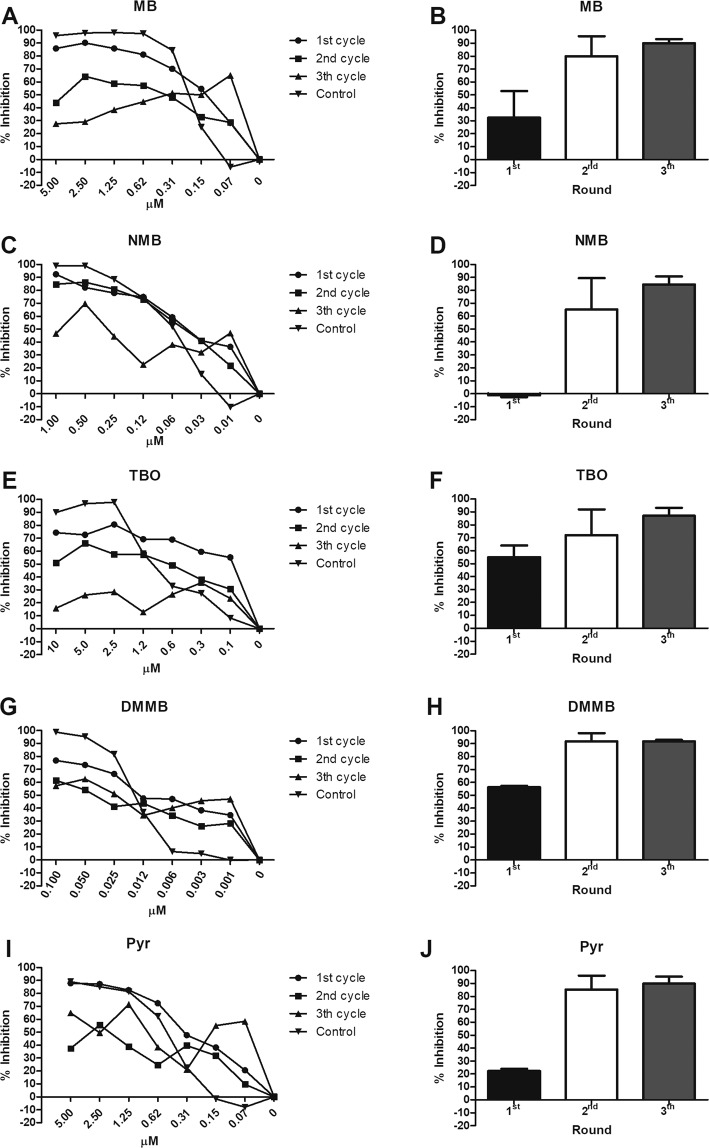
Resistance induction in *N. caninum* cultures. *N. caninum* tachyzoites (NcLacZCAT) were inoculated in Vero cells (5 × 10^5^/well) and treated with crescent concentrations of MB, NMB, TBO and DMMB and Pyr for 72 h, 37 °C, 5% CO_2_, in three consecutive rounds (the compound concentration in the first, second and third rounds was IC_50_, 2 × IC_50_, 4 × IC_50_, respectively). After each round, the samples were pooled, washed and applied for parasite burden evaluation, proliferation assays (in 96 well plates, as performed in item 3.3) and inoculated in a fresh Vero culture for the subsequent round. The proliferative assays and parasite burden evaluation were performed using CPRG assay and compared to the non-selected controls. (**A,C,E**), (**G**,**I**) represent the inhibitory curves of tachyzoites selected with IC_50_, 2 × IC_50_, 4 × IC_50_ (first, second and third round, respectively) and incubated with serial dilutions of phenothiazinium dyes or Pyr. (**B,D,F,H**) represent the parasite burden (percentage of inhibition compared to the non-treated control) after each round of selection. As a control, non-selected parasites were incubated under the same conditions for all evaluations.

All analyzed compounds induced parasite resistance after three rounds of selection (Fig. 2A,C,E,G,I), despite the different capacities to inhibit the tachyzoites proliferation (Fig. 2B,D,F,H,J). After the first or the second rounds of selection, each compound demonstrated different patterns of inhibition and resistance. The highest concentration of dyes (MB, NMB, TBO, and DMMB) applied in non-selected parasites (control curves, inverted triangles) inhibited > 90% of the parasite proliferation, whereas Pyr inhibited ~90%, as previously described^9,27^. MB induced a crescent parasite resistance after the successive rounds of selection (Fig. 2A), the same was observed for TBO (Fig. 2E) and DMMB (Fig. 2G). After the first, second and third rounds of selection with MB, ~ 85%, 60% and 30% of parasites were inhibited at higher doses, respectively (Fig. 2A). TBO and DMMB induced different levels of resistance, despite the high capacity of inhibition after each round (Fig. 2F,H). As observed for MB, the parasites selected with TBO demonstrated a high level of resistance: only 30% of parasites were inhibited by the dye after the third round. On the other side, DMMB inhibited > 55% of parasites even under the third round of selection. NMB showed the lower capacity to inhibit and induce parasite resistance among the analyzed compounds (Fig. 2C,D). The parasites selected with the IC_50_ and 2 × IC_50_ of NMB maintained a similar susceptibility to the dye compared to the non-selected counterparts (Fig. 2C).

Compared to the phenothiazinium dyes (excepting NMB), Pyr demonstrated a delayed resistance induction (Fig. 2I), which might be related to a distinct mechanism of action, known to be based on the inhibition of the DHFR enzyme^37^. Due to the single target for action, only part of the DHFR machinery is inhibited by Pyr at IC_50_ concentration, maintaining a residual folate metabolism, decreasing the selective pressure. The folate metabolism failure induced in higher Pyr concentrations (> 2 × IC_50_) probably improves the selective pressure, generating resistant parasites. On the other side, phenothiazinium dyes have multiple targets of inhibition in Apicomplexa, as demonstrated in *Plasmodium*^33^. Although the complete mechanism of action of MB and analogs in *Plasmodium* is unclear, the dye inhibits the Glutathione Reductase enzyme^38^, the polymerization of heme into hemozoin^39^ and also acts as a subversive substrate of antioxidant disulfide reductases^40^. Nevertheless, future assays related to the mechanism of action of dyes in *N. caninum* will be mandatory to elucidate the lower inhibitory/resistance induction of NMB compared to MB, TBO, and DMMB. Once all tested compounds induced parasite resistance, the use of single-drug therapies is potentially unfeasible for *N. caninum* using phenothiazinium dyes or Pyr. Indeed, for *T. gondii*, the use of drug combinations is a current and efficient strategy to minimize parasite resistance and recrudescence^41^.

### Clearance of parasites

The phenothiazinium dyes demonstrated different patterns for the *in vitro* clearance of *N. caninum*. TBO was excluded from the clearance assay due to its high inhibition dose: a toxic effect to the host cells would affect the interpretation of the results. For example, concerning the toxic values in cells (CC_50_), the concentrations applied in clearance assays (4 × IC_50_) were lower for MB (1.6 μM), NMB (0.2 μM) and DMMB (0.08 μM) compared to TBO (~ 7.5 μM). Moreover, the value of CC_50_/4 × IC_50_ for TBO was 3.3 whereas, for MB, NMB and DMMB were 39, 109.4 and 106.5, respectively.

**Figure 3:**
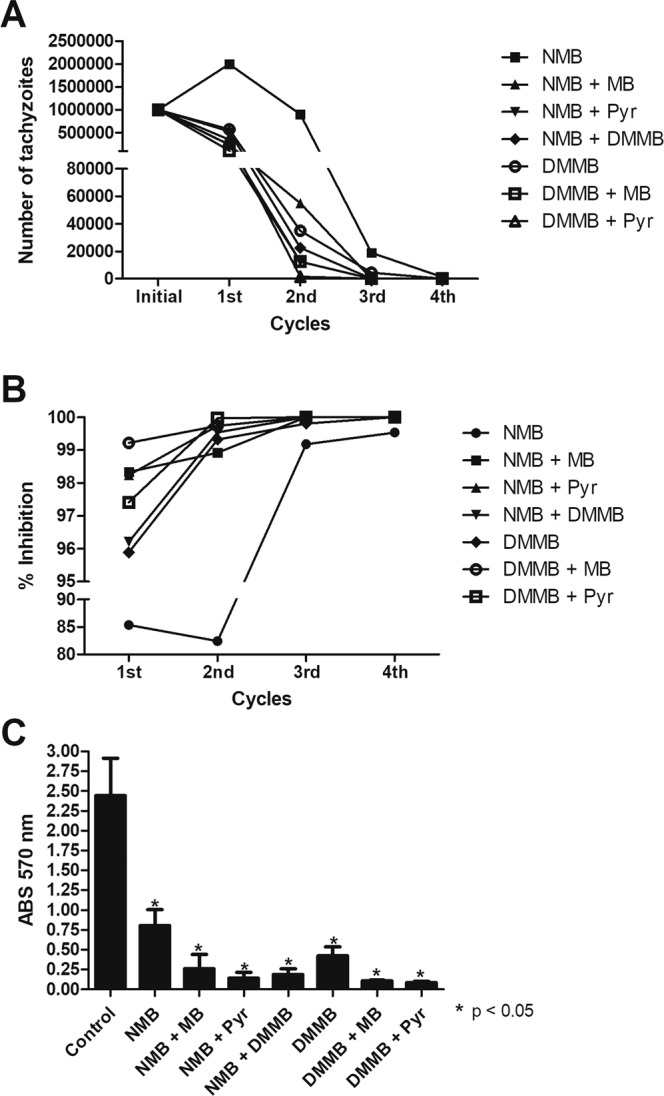
Clearance of *N. caninum* in Vero cells. Purified tachyzoites NcLacZCAT (1 × 10^6^/well) were incubated with 4 × IC_50_ of NMB, DMMB and combinations of these compounds with MB or Pyr for 72 h, at 37 °C, with 5% CO_2_. The extra-cellular tachyzoites were counted and re-incubated under the same conditions for four consecutive rounds. After each cycle, the percentage of parasite clearance was calculated. In the fourth cycle, the final parasite burden was detected after incubation with CPRG for 24 h. (**A**) The absolute number of tachyzoites after each cycle; (**B**) Percentage of clearance after each cycle; (**C**) Final parasite burden measured by CPRG reaction (24 h of incubation).

As observed previously for MB^27^, DMMB and NMB eliminated all extracellular tachyzoites after the fourth cycle (Fig. 3A). However, the tachyzoite clearance rate of NMB was delayed compared to DMMB. Whereas DMMB eliminated the tachyzoites above 95% in all cycles; NMB diminished the parasite burden in 85% and 82% after the first and second cycles, respectively (Fig. 3B). The combinations improved the capacity of DMMB and NMB, eliminating all extracellular tachyzoites after the third cycle. Among the compound combinations tested, all cycles reached over a 96% clearance rate. The CPRG assay, performed after the fourth cycle, indicated that NMB exhibited a higher *N. caninum* infection rate when compared to DMMB, demonstrating a diminution of the parasite burden in 68% and 79% respectively (Fig. 3C). No toxic effects were observed in Vero cells treated with compounds or combinations (data not shown). The compound combinations improved the parasite clearance; however, a different pattern was observed compared to the proliferation assays. The NMB + MB combination, synergistic in the proliferation assays, was inferior in comparison to the MB + DMMB combination. Moreover, all combinations of phenothiazinium dyes with Pyr demonstrated a clearance improvement compared to the single molecules (Fig. 3C), despite the antagonism observed in proliferation assays. Our previous work indicated a high capacity for parasite clearance under Pyr incubation, which was improved after combination with MB^27^. Moreover, we also observed the low *N. caninum* clearance capacity of Artemisinin *in vitro*, demanding the use of drug combinations^9^. Artemisinin is a classic example of drug resistance induction in *Plasmodium*. Thus, Artemisinin-based Combination Therapy (ACT) is strongly recommended for malaria treatment due to the emergence of Art-resistant parasites^42^. Likewise, a similar approach may be considered for *N. caninum*. Assays related to the mechanism of action of phenothiazinium dyes and Pyr are necessary, as there is a clear difference when compounds are tested in proliferation and clearance assays. Moreover, an adequate description of the mechanism of action of phenothiaziniums is also important to establish future formulations against neosporosis, prepared to act in a specific phase of the disease (acute, chronic and congenital).

### Phenothiazinium dyes increase the ROS levels in tachyzoites

**Figure 4:**
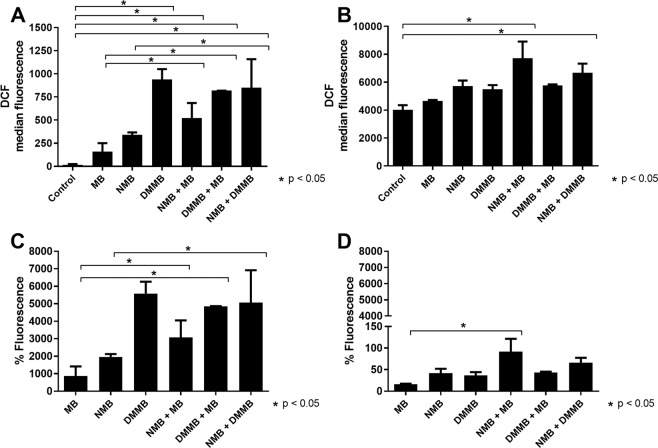
ROS production in tachyzoites and Vero cells incubated with MB, NMB, DMMB, and combinations. Tachyzoites (**A,C**) and Vero cells (**B,D**) were incubated with dilutions (4 × IC_50_) of NMB, MB, DMMB and its combinations (4 × IC_50_) for 30 min, 37 °C, 5% CO_2_. After the incubation, parasites or cells were washed with PBS and incubated with DCFDA (Sigma) for 15 min, 37 °C, 5% CO_2_ in the dark. Samples were analyzed in a BD FACS-Canto cytometer and the fluorescence intensity median achieved (**A,B**). The percentage of fluorescence was also calculated, compared to the no compound control (**C,D**). *p < 0.05.

Phenothiazinium dyes have the potential to elevate ROS in several cellular models^34,43–45^. However, the inhibitory concentrations for yeast, bacteria or mammalian cells are usually above 1 μM^35,46^, whereas against *N. caninum* the oxidant effects were observed at nanomolar levels (Fig. 4A), except for TBO. The effects of TBO in parasites and host cells may be probably conflicting due to the higher SI (13.6) compared to MB (179.1), NMB (456.1) and DMMB (448.6).

For *N. caninum*, NMB, DMMB and MB combinations resulted in a similar ROS level when compared to single compounds (Fig. 4A). The higher efficacy of phenothiazinium dye combinations compared to the single regimens in proliferation, clearance and *in vivo* assays do not directly correlate to ROS production. Whilst the dye combinations improved the anti-proliferative pattern, clearance rate and mice protection in comparison to the single treatments, ROS production was similar when these treatments were compared. Thus, novel approaches will be designed to elucidate the mechanisms for the compound synergism at a molecular level. Compared to single compounds, the combination of MB and NMB increased the ROS production in Vero cells significantly (Fig. 4B), but proportionally lower than observed in *N. caninum* (Fig. 4C,D). The compounds elevated the fluorescence percentage above 2000% in *N. caninum*, whereas this reached 163% against Vero cells (Fig. 4C,D), indicating different compound susceptibilities between parasite and host cell. Indeed, there is a relation between ROS elevation and inhibition of proliferation in *N. caninum* (not observed in Vero cells) when incubated with phenothiazinium dyes at 4 × IC_50_. Vero cells incubated with tested compounds at 4 × IC_50_ doses proliferate normally as verified in compound-free controls (data not shown).

### Dyes decrease the mitochondria potential in cultures

**Figure 5:**
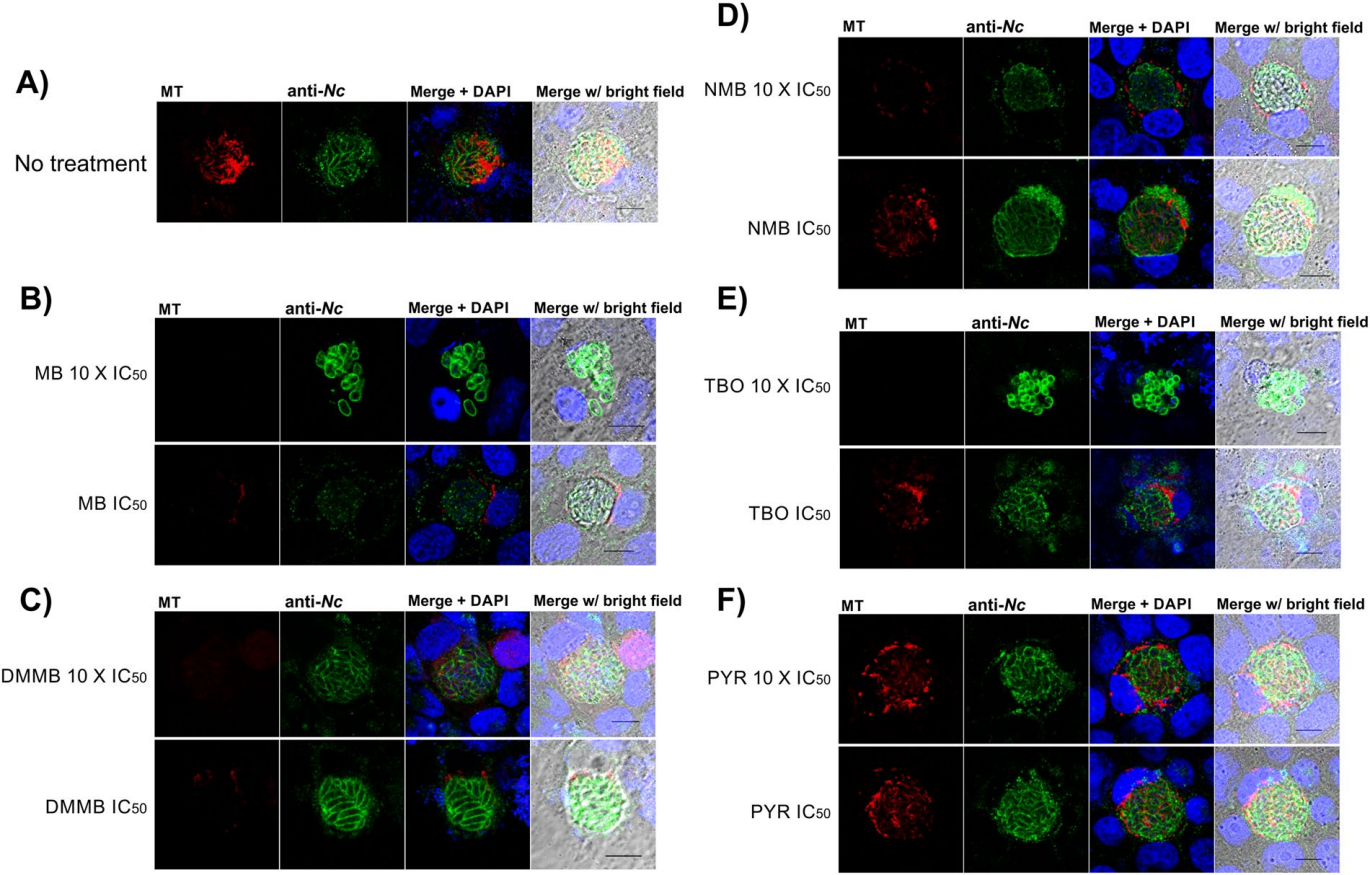
Effects of dyes in parasitophorous vacuoles and mitochondria. Tachyzoites (2 ×10^5^/well in 24 well plates) were incubated in Vero cell monolayers and incubated for 48 h, 37 °C, 5% CO_2_. The cultures were treated with the IC_50_ and 10 × IC_50_ of MB, NMB, TBO, DMMB and Pyr for 24 h, 37 °C, 5% CO_2_. Similarly, non-infected Vero cell cultures were incubated with dyes and Pyr under the same conditions. After washing, fixation, blocking and labeling with anti-*N. caninum* serum (Alexa 488, ex/em: 490/525), Mitotracker (ex/em: 579⁄599) and DAPI (ex/em: 358⁄461), the cultures were analyzed using confocal microscopy. The figures were composed by MT (Mitotracker), anti-Nc (anti-*N. caninum* serum), merge DAPI (Mitotracker + anti-Nc + DAPI) and merge w/ bright-field (Mitotracker + anti-Nc + DAPI + bright field) images. (**A**) Non-treated control; (**B**) MB treated; (**C**) DMMB treated; (**D**) NMB treated; (**E**) TBO treated; (**F**) Pyr treated. Bars indicate 10 μm.

The incubation of *N. caninum* infected or non-infected cells with phenothiazinium dyes demonstrated a different pattern in mitochondrial potential compared to Pyr (Fig. 5). At 10 × IC_50_, all dyes decreased the Mitotracker incorporation, whereas no alteration was observed for the non-treated control and Pyr (Fig. 5A,F). Moreover, the reduction of the mitochondrial potential has an evident effect in proliferating parasites rather than host cells, as observed in proliferation and clearance assays (Table 1 and Fig. 3). Indeed, *N. caninum* and *T. gondii* attract mitochondria during the proliferation process^47–49^, a crucial step for parasite survival. Moreover, MB, NMB, TBO, and DMMB decrease the *T. cruzi in vitro* mitochondria potential, impairing the parasite proliferation^50^. At IC_50_ concentrations, NMB and TBO (Fig. 5D,E) exhibited a similar pattern compared to Pyr or non-treated controls (Fig. 5A,F), corroborating the lower efficacy of the dye in clearance and resistance assays. Dyes in higher concentrations (MB and TBO at 10 × IC_50_) altered the parasite organization in vacuoles, indicating a widespread effect in the parasite/vacuole structure (Fig. 5B,E). On the other side, no effects in the cell structure were observed in cultures incubated with the compounds under the same conditions (Fig. 5). Thus, there are specific and general effects of dyes in vacuoles, dependent on the compound structure and/or concentration. For the complete elucidation of the mechanism of action of phenothiazinium dyes and Pyr in *N. caninum*, novel strategies related to mitochondria activity and parasite/vacuole structure will be adopted in future studies.

### Effects of phenothiazinium dyes in an *in vivo* model

The clearance assay is a more suitable method to guide a rational strategy for the treatment of neosporosis in animal models. The clearance assay reveals the elimination of parasites from cell culture, whereas proliferation measures the capacity of tachyzoite multiplication in the presence of the compound. Therefore, the *in vitro* clearance assay is similar to *in vivo* chemotherapy, since both methodologies are based on the parasite elimination from a host. Thus, mice infected with *N. caninum* were treated with DMMB alone or in combination with MB or Pyr, which demonstrated the highest in *in vitro* clearance rates (Fig. 3). TBO and NMB were excluded from the *in vivo* assays due to the higher SI compared to MB and DMMB and due to the lower clearance and mitochondria depolarization capacity, respectively. We based the *in vivo* parameters on articles that analyzed the effects of MB in models using mice^36,51–53^. MB was tested in different doses (2.5 to 2000 mg/Kg) with deleterious effects usually observed in long term treatments (five weeks) using doses >100 mg/Kg/day^53^. However, no studies have ever been performed for assessing the *in vivo* toxic effects of DMMB, demanding complementary toxicological assays. In a murine model of malaria, it was estimated that a regimen using MB 45.77 mg/Kg/day eliminates 90% of *Plasmodium berghei*^36^. Moreover, the effectiveness of intraperitoneal MB (50 and 100 mg/Kg/day) to control the blood stage of *Plasmodium yoelii* in BALB/c mice was also demonstrated^54^. Thus, for the initial treatment of animals acutely infected with *N. caninum*, a dose of 50 mg/Kg/day (MB and/or DMMB) represented a rational choice. We used cerebral neosporosis as a first approach once the main features of the compounds (mortality, morbidity, parasite burden, and toxicity) may be assessed before the use of more complex models.

**Figure 6:**
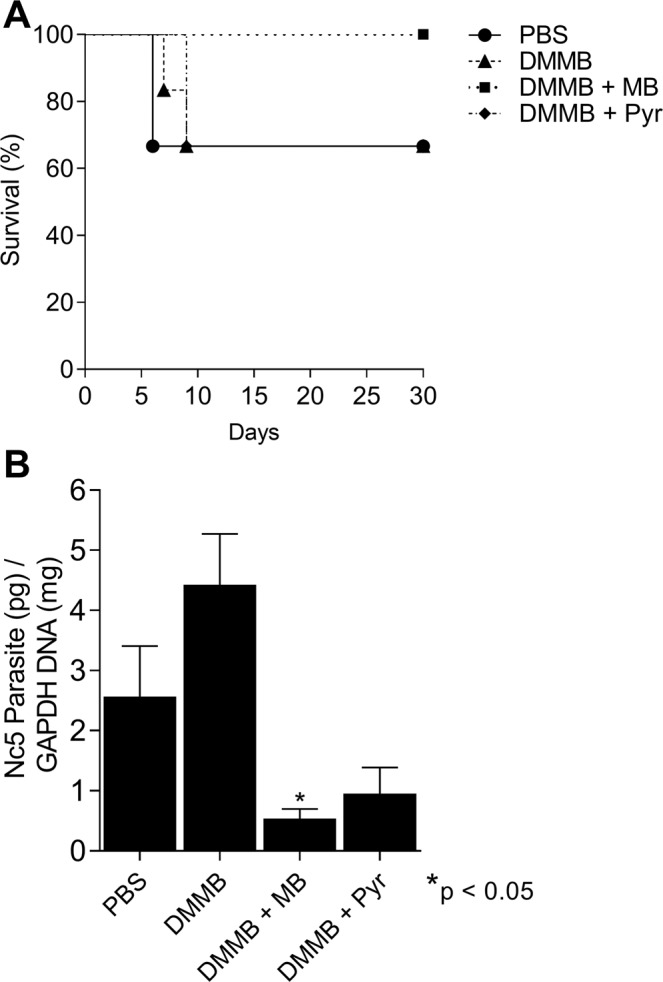
*In vivo* evaluation of DMMB and combinations. C57Bl/6 mice were infected with 1 × 10^7^ *N. caninum* tachyzoites (NcLiv) and after 12 h treated with five single doses of DMMB (50 mg/Kg/day) and its combinations with Pyr (10 mg/Kg/day) or MB (50 mg/Kg/day) for five days. The control was composed of infected animals treated with PBS. The animals were observed for 30 days and the surviving rate (**A**) and cerebral parasite burden (**B**) determined. * represent p < 0.05 compared to the PBS group.

On the sixth day after *N. caninum* infection, 2 out of 6 animals treated with PBS died. Under treatment with DMMB 1 out of 6 died after the seventh and eighth days. The DMMB + Pyr combination demonstrated a similar result, with 2 animals succumbing after the eighth day. However, all animals (6/6) were protected by the combination of DMMB with MB (Fig. 6A) and no evident clinical signs (ruffled coat, apathy, walking disorders, rounded back and paralysis) were observed. Despite similar protection of animals treated with DMMB and the combination DMMB + Pyr, the cerebral parasite burden from the surviving animals was different. The ratio of parasite/host cell DNA in animals treated with DMMB and DMMB + Pyr combination was 4.43 and 0.95 respectively. The DMMB + MB combination reached 0.53, whereas for PBS control the relation was 2.56 (Fig. 6B). The combinations demonstrated superior efficacies compared to DMMB applied alone, elevating the surviving rate and/or decreasing the parasite burden. The efficacy of DMMB alone for the treatment of infected animals demonstrated the limitations of single-compound based chemotherapies. Drugs usually have few therapeutic targets, and this is usually insufficient against multiple evasion mechanisms in protozoans such as *N. caninum* or *T. gondii*^55^. Moreover, parasite resistance to Pyr has been achieved in *N. caninum* and *T. gondii* in culture^56,57^. Surprisingly, compounds from the same class (DMMB and MB) demonstrated superior performance compared to combinations based on compounds belonging to different categories (DMMB and Pyr), indicating possibly more than one mechanism of action among phenothiazinium dyes against *N. caninum*. Once the synergistic phenomena observed in the proliferation (NMB + MB) and *in vivo* approaches (DMMB + MB) have been not observed in Apicomplexa, further studies will be required. No toxic effects were observed (apathy, weight loss, behavior) in treated animals for 30 days. However, complementary assays such as dose-dependent treatment, use of alternative routes of administration and/or infection will be required to determine a safe application level of these compounds/combinations for clinical use. The data from the acute model of infection, as applied in this study, is an initial approach and usually will be useful for future testing in chronic and congenital models.

The combinations of MB with NMB or DMMB demonstrated synergism *in vitro* or *in vivo* respectively, which is usually observed when effective compounds from different families are combined^58^. Thus, further work will be performed to investigate the common and exclusive targets of phenothiazinium dyes. Moreover, the effectiveness, pharmacodynamics, half-life, and clearance of each compound (and the combinations) are fundamental points for the design of future clinical strategies. Despite the absence of toxic effects in the host cell in all assays using MB, NMB and DMMB, complementary strategies to clarify the side effects of these compounds and formulations will be demanded. Since there is no data concerning the use of phenothiazinium dyes for the treatment of food animals, our study describes a new potential form to control neosporosis. The use of low-cost compounds, such as phenothiazinium dyes against neosporosis, represents a suitable form for livestock management, improving the productivity of the cattle and impacting the economy of beef and dairy industry worldwide.

## Methods

### Neospora caninum

*N. caninum* Nc1 expressing β-galactosidase (NcLacZCAT)^59^ was used in proliferation and clearance assays. For oxidative stress assays, Nc1 purified tachyzoites (using 5 μm syringe filtration) were employed. In the *in vivo* model of infection, the virulent *N. caninum* Liverpool strain (NcLiv) was used. NcLiv was cultivated in Vero cell monolayers for 30 passages when it was inoculated in C57Bl/6 mice to improve its virulence. The *in vivo* assay was performed using NcLiv cultures at the 20^th^ passage.

### Pyrimethamine and phenothiazinium dyes

Methylene Blue ([7-(dimethylamino)phenothiazin-3-ylidene]-dimethylazanium;chloride, catalogue number: 1428008), New Methylene Blue (ethyl-[7-(ethylamino)-2,8-dimethylphenothiazin-3-ylidene]azanium;chloride, catalogue number: R313718), Toluidine Blue O ((7-amino-8-methylphenothiazin-3-ylidene)-dimethylazanium;chloride, catalogue number: T3260), 1,9 – Dimethyl-Methylene Blue ([7-(dimethylamino)-1,9-dimethylphenothiazin-3-ylidene]-dimethylazanium;chloride, catalogue number: 341088) and Pyrimethamine (5-(4-chlorophenyl)-6-ethylpyrimidine-2,4-diamine, catalogue number: 1589007) were purchased from Sigma-Aldrich and diluted (5 mg/ml each) in PBS (MB, NMB, TBO and DMMB) or DMSO (Pyr) for *in vitro* use. For *in vivo* application, DMMB, MB and Pyr were diluted (2.5 mg/ml) in PBS.

### Proliferation assay

The proliferation assay was performed as described in^27^. Briefly, purified *N. caninum* tachyzoites were distributed (1 × 10^3^/well) in a Vero cell culture in a 96 well plate and incubated for 2 h to allow cell invasion. After the invasion process, seven NMB, TBO or DMMB dilutions (starting from 0.25 μM, 20 μM and 0.12 μM respectively) were added, in triplicate, to the infected cells and the plates were incubated for 72 h, at 37 °C with 5% CO_2_. For the evaluation of the drug combinations, after parasite invasion, seven fractional inhibitory concentrations (2 × IC_50_, 1.6 × IC_50_, 1.3 × IC_50_, IC_50_, 0.7 × IC_50_, 0.5 × IC_50_, 0.2 × IC_50_), of each compound alone or combined were applied on proliferating tachyzoites under the same conditions. The 2 × IC_50_ concentrations for MB, Pyr, NMB, and DMMB were 0.7, 0.7, 0.125, and 0.05 μM, respectively. The drug combination was composed of a solution containing an equal concentration of each compound. As a control, the same concentration of every single compound was used. After incubation, the wells were washed with PBS and lysed with 125 μl CPRG lysis buffer (100 mM 4- (2-hydroxyethyl)-1-piperazineethanesulfonic acid, pH 8.0; 1 mM CaCl_2_; 1% Triton X-100, 0.5% SDS; 5 mM DTT) for 1 h at 50 °C. The lysates were incubated with 125 μl of CPRG buffer (5 mM CPRG, 5 mM 2-mercaptoethanol in PBS) for 1 h and the plates read at 570 nm in an ELISA reader (Sunrise, Tecan). Three independent assays were performed.

### Cytotoxicity

The deleterious effects of the phenothiazinium dyes to the host cells were evaluated by MTT assay^60^ adapted for Vero cells^27^. Briefly, 5 × 10^3^ Vero cells/well were cultivated in 96 well plates until cell confluence. The cells were cultured with dilutions of NMB, TBO, and DMMB (starting from 100 μM) for 72 h, at 37 °C, with 5% CO_2_. The treated wells were incubated with MTT solution (500 μg/ml) for 4 h, 37 °C, 5% CO_2_ followed by formazan dilution within acidified isopropanol (HCl 50 mM). The plates were read at 570 nm in an ELISA reader (Sunrise, Tecan) and cytotoxicity estimated as previously described^27^. Three independent assays were performed.

### Resistance assay

The resistance assay was schematically described in Supplementary Fig. 1. NcLacZCAT tachyzoites (5 × 10^5^/well) were distributed in Vero cell monolayers (cultivated in 24 well plates) and incubated for 2 h, 37 °C, 5% CO_2_ for cell invasion. After the invasion, the cultures were washed with PBS and treated with the IC_50_ concentration of phenothiazinium dyes and Pyr (0.5 μM, 0.6 μM, 2 μM, 0.025 μM and 0.5 μM of MB, NMB, TBO, DMMB, and Pry, respectively) for 72 h, 37 °C, 5% CO_2_. The control was incubated with RPMI with 0.05% DMSO (similar to Pry treated samples), under the same conditions. The treated samples and the non-treated control were pooled in 15 mL tubes, washed with PBS and resuspended in 6 ml of RPMI without phenol red. The suspension was divided into three parts. The first part of the suspension (1 ml/tube) was centrifuged (3 minutes, 2500 g) and applied for the parasite burden evaluation by CPRG assay (24 h of incubation at 37 °C). Likewise, the second part of the suspension was applied in proliferation assays as described in item 3.3. The selected parasites (125 μl/well) were inoculated in duplicate in 96 well plates containing Vero cell monolayers. The plates were incubated for 2 h, 37 °C, 5% CO_2_ for cell invasion and treated with seven serial dilutions of dyes and Pyr. The higher concentrations of MB, NMB, TBO, DMMB, and Pry applied in proliferation assays were 5 μM, 1 μM, 10 μM, 0.1 μM, and 5 μM respectively. The plates were incubated for 72 h, 37 °C, 5% CO_2_ and analyzed by CPRG assay, as described in item 3.3. From CPRG assays, the percentage of inhibition in comparison to the non-treated samples was calculated and plotted. Finally, the third part of the suspension of 3 ml (1 ml/well) was inoculated in fresh Vero cell monolayers, as performed in the first round of selection. In the second round of selection, the cultures were treated with 2 x IC_50_ of phenothiazinium dyes and Pyr. After the second round of selection, the samples were analyzed as described for the first one. Finally, the third round of selection was also performed under the same conditions using 4 × IC_50_ of phenothiazinium dyes and Pyr and analyzed as previously described. Two independent assays were performed.

### Clearance assay

Clearance assays were performed as previously described^27^. Briefly, purified tachyzoites (NcLacZCAT, 1 × 10^6^/well) were allowed to invade Vero cells monolayers, cultured in 24 well plates, for 2 h, at 37 °C, with 5% CO_2_. After the invasion, NMB, DMMB, and combinations of these compounds with MB or Pyr (4 × the IC_50_ of each one) were added and incubated for 72 h, at 37 °C, with 5% CO_2_. After this, the cultures were detached and the extracellular tachyzoites counted in a hemocytometer. The remaining tachyzoites were incubated in a new Vero cell culture under the same conditions. Four consecutive cycles of incubation were performed and the inhibition percentage and the absolute number of tachyzoites were calculated. Finally, the final parasite burden was measured after incubation with CPRG for 24 h as described in the proliferation assay.

### Evaluation of oxidative stress in tachyzoites

Tachyzoites (Nc1 strain) and Vero cells were used in oxidative stress assays. Purified tachyzoites (1 × 10^6^/tube) or cells (a confluent culture in a 24 well plate, in duplicate), in RPMI without phenol red, were incubated with 4 × IC_50_ of MB (1.6 μM), 4 × IC_50_ of NMB (0.25 μM) and 4 × IC_50_ of DMMB (0.1 μM) for 30 min at 37 °C with 5% CO_2_. Additionally, these dyes were combined (MB + NMB; MB + DMMB; NMB + DMMB) and applied to parasites or cells under the same conditions. After the incubation, tachyzoites, and cells were washed twice with PBS and processed for flow cytometry analysis. Cells were incubated with 0.05% trypsin in PBS (Life Technologies) supplemented with 2 μM DCFDA (Sigma) for 30 min, at 37 °C, with 5% CO_2_ in the dark. Tachyzoites were incubated under the same conditions; however without a trypsinization step. The median fluorescence intensity (from gates representing cells or tachyzoites) was measured in a BD FACS-Canto (BD Biosciences) flow cytometer (Ex.488/Em.535 nm) with the FACSDiva (BD) 6.1.3 software. The fluorescence percentage was calculated in relation to the non-treated group. Two independent experiments were performed for each assay.

### *N. caninum* structure and mitochondrial potential in parasitophorous vacuoles

For confocal analysis, tachyzoites were inoculated (2 × 10^5^/well) over Vero cell monolayers in 24-well plates with coverslips at the bottom and incubated during 48 hours for invasion and proliferation. After incubation, the cultures were treated with IC_50_ and 10 × IC_50_ of MB (0.5 μM / 5 μM), NMB (0.06 μM / 0.6 μM), TBO (2 μM / 20 μM), DMMB (0.02 μM / 0.2 μM) and Pyr (0.5 μM / 5 μM) for 24 h at 37 °C and 5% CO_2_. The supernatants were discarded and 400 nM Mitotracker (Mitotracker Red CMXRos, Invitrogen, Thermo Fisher Scientific, OR, USA) were added to the wells and incubated for 20 minutes at 37 °C and 5% CO_2_. The supernatants were discarded and the wells were washed twice with PBS. Then, the cells were fixed with 3.7% formaldehyde, followed by permeabilization (0.2% Triton X-100) and blocking (2% bovine serum albumin; 20 mM glycine). The nuclei were observed using 300 nM 4′,6-diamidino-2-phenylindole (DAPI, Molecular Probes, Life Technologies, OR, USA). *N. caninum* was detected with serum *anti-N. caninum* (1:1,000) and anti-mouse conjugated with Alexa 488 (5 µg/ml; Molecular Probes, Life Technologies, OR, USA). The anti-*N. caninum* serum was generated in BALB/c animals (CEUA-FCFRP, process 17.5.278.60.8), detailed in the Supplementary Material (Supplementary Material 2). The coverslips were mounted on a slide and sealed with liquid blocker (Super PAP Pen mini, Doido Sangyo Co. Ltd., Tokyo, Japan). The slides were observed in a TCS-SP8 AOBS (Leica Microsystems), using a 63 X objective and processed by ImageJ software (National Institute of Health, USA). The excitation/emission wavelengths for Mitotracker, DAPI and Alexa 488 were 579⁄599 nm, 358⁄461 nm, and 490/525 nm, respectively.

### *In vivo* neosporosis model for treatment

For the *in vivo* model of infection, six-week-old male C57Bl/6 mice were used (6 animals/group), according to the local Animal Ethics Committee, approved by the animal research committee at Universidade Federal de Uberlândia (UFU) (Comitê de Ética na Utilização de Animais da UFU – CEUA/UFU), under protocol number 109/16. The treatment strategy was based on previous studies performed with *N. caninum*^4,13,14,17,61^. The animals were intraperitoneally (i.p.) infected with 1 × 10^7^ NcLiv tachyzoites at day 0 and after 12 h the animals were treated with each compound and their combinations. Single doses of DMMB (50 mg/Kg/day, i.p.) or combinations of this dye with Pyr (10 mg/Kg/day, oral gavage) or MB (50 mg/Kg/day i.p.) were administered during five days. A dose of Pyr was chosen which is insufficient to cure the animal against the disease caused by *T. gondii*^62^ and was suitable as a parameter for the drug combination studies. The non-treated control received only PBS. Animals were observed daily for clinical signs (ruffled coat, apathy, walking disorders, rounded back, and paralysis), and mortality during 30 days post-infection. After 30 days of challenge, surviving animals were euthanized and the brain tissues (20 mg) were collected for parasite quantification using real-time PCR (SYBR green detection system; GoTaq qPCR Master Mix, Promega), as previously described^63,64^. The genomic DNA was extracted using (Wizard SV Genomic DNA kit, Promega) and the concentration normalized to 40 ng/μl (Nanodrop Lite, Thermo Scientific). *N. caninum* DNA was amplified (StepOnePlus, Thermo Scientific) using primers Nc5 (sense 3′ GCTGAACACCGTATGTCGTAAA 5′; antisense 3′ AGAGGAATGCCACATAGAAGC 5′) and normalized using primers for mouse GAPDH (sense 3′ CTCGTCCCGTAGACAAAATGG 5′; antisense 3′ AATCTCCACTTTGCCACTGCA 5′). The cerebral parasite burden was calculated by interpolation from a standard curve established from serial dilutions of DNA extracted from tachyzoites.

### Statistical analyses

The percentages of proliferation and toxicity were calculated using the mean absorbance of the compound-free control and the absorbance from each treatment as described previously^27^. The IC_50_ and the combination index (CI) were obtained using Compusyn software (http://www.combosyn.com/) and the selective index (CC_50_/IC_50_) was also determined. From CI values, synergism (CI < 1) and antagonism (CI > 1) interactions were generated^65^. For clearance, oxidative stress and *in vivo* (parasite burden) assays, data were analyzed by one-way ANOVA followed by a Tukey’s *post hoc* test (compared to non-treated groups) on GraphPad 5.0 software.

## Supplementary information

Supplementary Information.
