# Supplementary material for: Inhibitory action of phenothiazinium dyes against Neospora caninum
Source: Sci Rep. 2020 May 4;10:7483. doi: 10.1038/s41598-020-64454-x (PMC7198568; doi:10.1038/s41598-020-64454-x)
Supplement: Supplementary file 1 — Supplementary Information. [file 41598_2020_64454_MOESM1_ESM.docx]

**Inhibitory action of phenothiazinium dyes against *Neospora caninum***

Authors: Luiz Miguel Pereira; Caroline Martins Mota; Luciana Baroni; Cássia Mariana Bronzon da Costa, Jade Cabestre Venancio Brochi; Mark Wainwright, Tiago Wilson Patriarca Mineo; Gilberto Úbida Leite Braga; and Ana Patrícia Yatsuda

**Supplementary material 1**

**Resistance assay scheme**

**
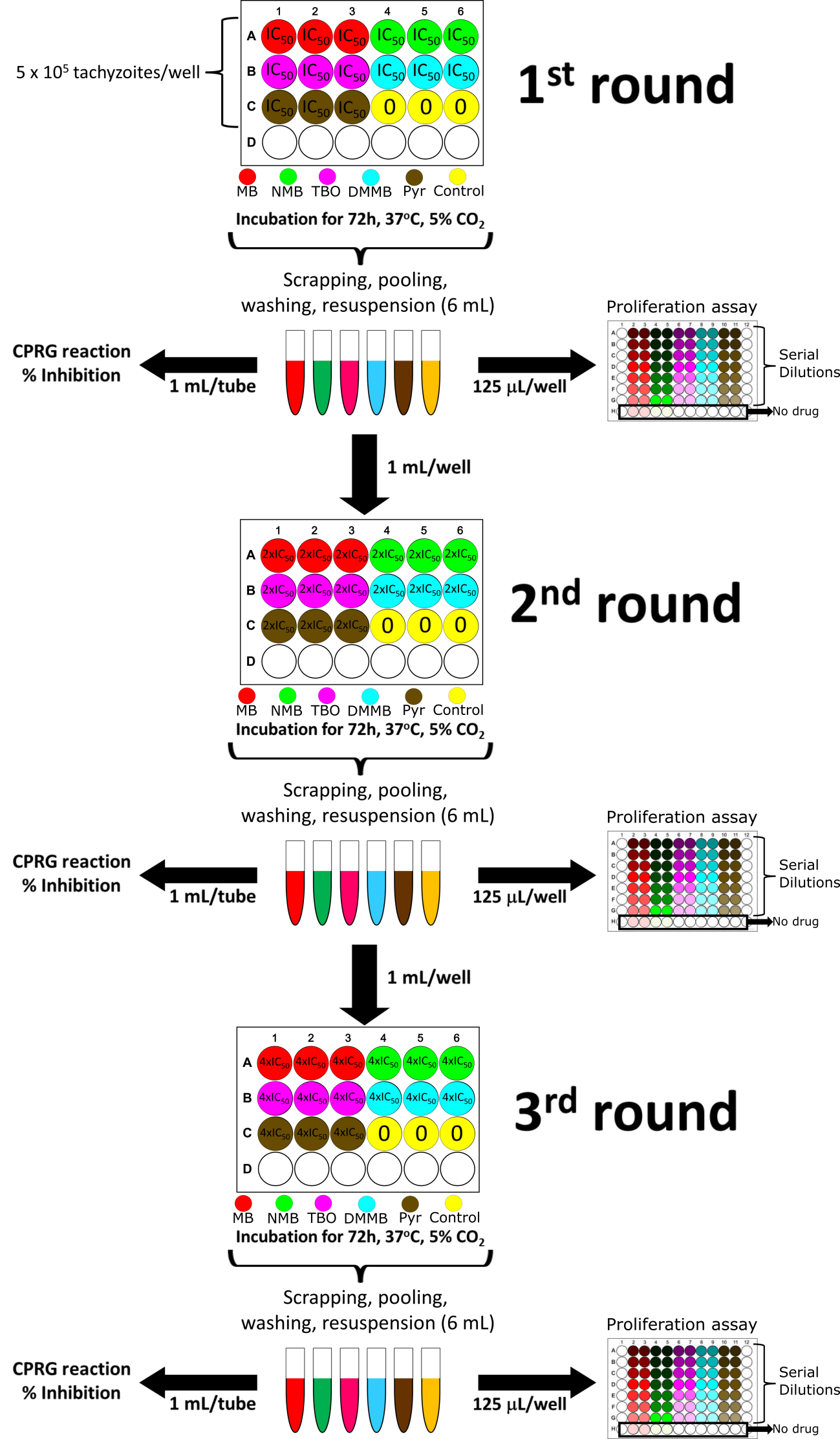
**

**Supplementary figure 1. Schematic description of the resistance assay procedure.** Nc-LacZ tachyzoites (5 × 10^5^/well) were distributed in Vero cell monolayers (in 24 well plates) and incubated with the IC_50_ concentration of MB, NMB, TBO, DMMB, Pyr for 72 h, 37^o^C, 5% CO_2_. The samples were pooled, washed and resuspended in 6 ml of RPMI. The suspension was divided into three parts: 1 ml for parasite burden evaluation by CPRG assay; 125 ml/well for proliferation assay and 3 ml (1 ml/well) for inoculation in a fresh 24 well plate (2^nd^ round) containing Vero cell monolayers. The proliferation assay was performed as described in item 3.3 and evaluated by CPRG assay. The samples applied for the second round were incubated with 2 × IC_50_ of phenothiazinium dyes and Pyr for 72 h, 37^o^C, 5% CO_2_ and processed as described in the first round. Finally, the third round of selection was performed using dyes and Pyr at 4 × IC_50_ under the same parameters of the first and second ones. For all assays, controls composed by non-treated cultures were applied and compared to the treated counterparts.

**Supplementary material 2**

**Generation of *N. caninum* anti-serum**

BALB/c mice were applied for *N. caninum* anti-serum production. Five male animals with 6 – 8 weeks were inoculated in intervals of 15 days with a *N. caninum* protein extract (50 μg/animal/boost). The *N. caninum* protein was composed of tachyzoites sonicated with urea 8M and mixed 1:1 with aluminum hydroxide (1:1, Alhydrogel 2%, Brenntag, Biosector A/S, Denmark). Four boosts were performed after animal anesthetized with ketamine (80 mg/kg) and xylazine (8 mg/kg) (Kawai et al., 2011). At the 15^th^ day after the fourth round of immunization, the animals were anesthetized in the same way and euthanized after exsanguination by cardiac puncture. The blood was centrifuged (1000 g, 5 minutes, 4^o^C) and the serum harvested and pooled. All animal experimental procedures were conducted following the Animal Research Ethics Committee of the School of Pharmaceutical Sciences of Ribeirao Preto from University of Sao Paulo (CEUA-FCFRP, process 17.5.278.60.8).

**Reference**

Kawai, S., Takagi, Y., Kaneko, S., Kurosawa, T., 2011. Effect of three types of mixed anesthetic agents alternate to ketamine in mice. Exp. Anim. 60, 481–7.
